# Childhood Cancer Survivors: An Overview of the Management of Late Effects

**DOI:** 10.3390/cancers15123150

**Published:** 2023-06-11

**Authors:** Maura Argenziano, Alessandra Di Paola, Francesca Rossi

**Affiliations:** Department of Woman, Child and General and Specialist Surgery, University of Campania “Luigi Vanvitelli”, Via Luigi De Crecchio, 4, 80138 Naples, Italy; maura.argenziano@unicampania.it (M.A.); alessandra.dipaola@unicampania.it (A.D.P.)

The collection of papers in this Special Issue entitled “Frailty in Pediatric and Young Adult Cancer Survivors: from bench to bedside” includes six interesting articles (five reviews and one single-center retrospective longitudinal cohort study) presented by expert researchers in the fields of oncology and pediatrics. The aim of these publications is to highlight the condition—in the majority cases, very disabling—displayed by childhood cancer survivors (CCS), as well as to propose new management perspectives to ameliorate the quality of life of these subjects. One of the greatest medical successes of recent decades is certainly the improvement of the survival rate of pediatric cancer: almost the 80% of children affected by cancer become long-term survivors, achieving 5-year survival, without any administered therapy [1]. Despite this encouraging observation, chronic late effects related to anticancer treatments continue to affect CCS [2], accelerating the physiological aging process (premature aging). Therefore, CCS prematurely experience age-related health diseases, leading to frequent hospitalization and early mortality in a general and systemic condition known as “frailty” [2,3]. In the elderly, frailty is physiologically influenced by lifestyle and genetic factors, while in young adult CCS, this condition is strongly related to the organ system damage caused by oncological therapies [3].

In particular, exposure to oncological therapies (mainly chemo- and radiotherapy) causes low-grade systemic inflammatory state onset, senescent cell accumulation, the increased production of reactive oxygen species (ROS), immune response activation and DNA mutations [4,5]. This global inflammatory condition is known as “inflamm-aging” to indicate the correlation between inflammatory processes—exacerbated in CCS subjects—and the observed pathological conditions displayed by young adult CCS even though they are characteristic of an elder age [6]. The inflamm-aging process involves any organ and tissue, and can lead to the development of osteoporosis (OP), infertility, metabolic disorders, and cardiovascular diseases [7,8,9]. As described in this Special Issue section, OP is the main bone disease worldwide, and it is a systemic disease characterized by bone tissue fragility and a high risk of fractures, due to bone mass accumulation deficits [10]. The process of bone mass accumulation particularly occurs in adolescence, but several factors—female sex, immobility, a lack of nutrition and also oncological therapy—could affect this process, contributing to OP onset. Indeed, in CCS subjects, the exposure to anticancer drugs is responsible for a lower bone mass accumulation, leading to an increased risk of developing OP in young adulthood. Among the most innovative therapeutic proposals for CCS affected by OP, there are cannabinoids, molecules able to stimulate cannabinoid receptor type 2 (CB2). This receptor is physiologically involved in bone metabolism, and its proper stimulation inhibits the erosive action of osteoclasts (OCs), thus limiting bone disruption. It is known that chemotherapy causes a very strong decrease in the CB2 expression level in OCs, highlighting the possibility to use it as a marker of frailty and also as a therapy target for CCS subjects affected by cancer-therapy-related OP [11]. Inflammation is also closely related to the onset and maintenance of cardiovascular diseases in CCS. Among these diseases, heart failure, coronary artery disease, stroke, arrhythmias, and valvular dysfunction are of major concern for long-term survivors of childhood cancer, and are attributable especially to anthracycline chemotherapy and chest-directed radiation therapies [12]. There are a few preclinical studies evaluating cardiotoxicity in growing and developing hearts, but taking into account inflammatory processes could also ameliorate this issue in CCS patients. Anti-inflammatory drugs can be considered promising therapeutical approaches to counteract hypertension and dyslipidemia, for example, two of the most important risk factors for cardiovascular disease occurrence [13].

Cancer therapies administered during childhood also affect human fertility, since they are responsible for direct and indirect negative effects on the gonads. In particular, in the literature, the role of alkylating agents and irradiation as the factors most responsible for a decrease in ovarian reserves is well documented [14]. In this collection, we can read the systematic review by Torella et al. describing the role of serum anti-Müllerian hormone (AMH) as a non-invasive ovarian reserve marker as well as a marker of the risk of premature ovarian insufficiency (POI) in CCS populations. After anticancer treatment, some girls present a decrease in AMH levels that can rise again two years after stopping therapy. Unfortunately, in some cases, this event is not reversible, and POI occurs, making it impossible to get pregnant. Here, the necessity of interventions, such as ovarian cryopreservation, to avoid reproductive issues is highlighted [15]. The authors of this review also analyzed the correlation between long-term reproductive sequelae experienced by female CCS and the type of cancer (i.e., thyroid cancer, neuroblastoma), describing the same necessity to follow-up patients during and after anticancer therapy administration for all different kinds of cancer. Another substantial burden of classical chemotherapy is neurotoxicity. Side effects affecting the central nervous system (CNS) can include cerebellar degeneration, myelopathy, and effects on vision, hearing or taste, and generally appear when drugs are intrathecally administered or used at high dosages [16]. Neurotoxicity seems to be exacerbated when chemotherapy and radiotherapy are combined. Even though there are no specific studies on CCS, in particular on pediatric populations, low-dose radiation exposure is known to cause long-term toxic effects on the brain, such as premature massive nervous cells senescence [17]. After radiation exposure, immunotherapy also seems to be responsible for neurotoxicity. Little is known about immunotherapy, maybe because it has only recently been included in anticancer therapeutic protocols, especially for hematological neoplasms. Blinatumomab administration and the chimeric antigen receptor (CAR) T-cell protocol are the main oncological treatments associated with neurotoxicity [18]. Despite the great advances made in the field of oncology, the related neurological issues are still not completely described and, as consequence, are not completely manageable. Currently they have a low incidence (about 5-10%) and are mainly reversible. Prevention and early recognition with a proper follow-up among relevant children are the vital strategies to preserve neurological functions in the long term. Other important issues analyzed in this Special Issue include the high morbidity and late mortality observed in CCS affected by hypothalamic gliomas. In particular, in the reported cohort study, 90 children diagnosed before their third birthday were studied in a 10-year follow-up period. Overall, 57.8% of these subjects experienced endo-metabolic dysfunction, such as obesity, and also neurobehavioral deficits [19]. These events are strictly related to injury derived from a hypothalamic tumor location, as well as adjuvant treatment strategies. Another aspect that should be analyzed—the last but not the least—is the risk that CCS will develop subsequent or secondary malignant neoplasms, namely malignancies that affect children with primary cancer but are histologically different from the original tumor. This “late effect” is more frequent in CCS than in adolescent and young adult cancer survivors, probably because children’s tissues and organs are more sensitive to chemotherapeutic drugs and radiation [20], and overall, they are more prone to develop a chronic state of inflammation that negatively alters tissue functionality. This problem has a high social, economic and medical impact, hence the necessity to increase the quality of oncological treatments, thus reducing late effects.

## Discussion

Certainly, the risk of developing late effects after cancer treatments is very high, and focusing studies on related immune system alteration, oxidative stress, cell senescence, and the overall inflamm-aging process could be an appropriate strategy to predict and prevent life-threatening effects affecting CCS subjects. The papers included in this collection greatly contribute to describing the problems affecting CCS, as well as proposing some intervention possibilities, thus providing a good future perspective for the management of CCS, including many years after originally stopping therapy.
